# PROPOSAL FOR A REHABILITATION PROTOCOL AFTER CALCANEAL TENDON RECONSTRUCTION: FROM THE IMMEDIATE POST-OPERATIVE PERIOD TO RETURN TO SPORTS PRACTICE

**DOI:** 10.1590/1413-785220253306e292035

**Published:** 2025-11-10

**Authors:** Flavia Cursino de Vicente, Georgia Melges de Souza, Cleidneia Aparecida Clemente, Perola Grinberg Plapler

**Affiliations:** 1 Universidade de Sao Paulo, Faculdade de Medicina, Hospital das Clinicas (HCFMUSP), Instituto de Ortopedia e Traumatologia, Sao Paulo, SP, Brazil.; 2 Faculdades Integradas de Santo Andre, Sao Paulo, SP, Brazil.

**Keywords:** Rupture, Achilles Tendon, Rehabilitation Protocols, Ruptura, Tendão Calcâneo, Protocolo de Reabilitação

## Abstract

The calcaneal tendon is the strongest tendon in the human body, and therefore the most commonly injured in the lower limbs. The aim of this study is to present a rehabilitation protocol, based on the literature, according to the physiology of tissue regeneration in the postoperative period of acute rupture of the calcaneal tendon, carried out through a bibliographic survey of the last 20 years and proposed by the Physiotherapy Service of the Institute of Orthopaedics and Traumatology of the Hospital das Clínicas of the University of São Paulo. The findings demonstrated that, despite the differences in surgical techniques, the proposed rehabilitation protocol presents minimal risk of damage to the surgical site. ***Level of Evidence III; Systematic Review***.

## RESUMO

O tendão calcâneo é o tendão mais forte do corpo humano, portanto o que apresenta maior prevalência de lesão nos membros inferiores. O objetivo desta pesquisa é apresentar o protocolo de reabilitação, embasado na literatura de acordo com a fisiologia de regeneração tecidual no pós-operatória do reparo da ruptura aguda de tendão do calcâneo, realizado através de levantamento bibliográfico dos últimos 20 anos e proposto pelo Serviço de Fisioterapia do Instituto de Ortopedia e Traumatologia do Hospital das Clínicas da Universidade de São Paulo. Onde ficou evidenciado que apesar das diferenças das técnicas cirúrgicas o protocolo proposto de reabilitação apresenta risco mínimo de dano ao sítio cirúrgico. ***Nível de Evidência III; Revisão Sistemática***.

## INTRODUCTION

The calcaneal tendon is a structure composed of type I collagen, proteoglycans, and elastin that converges from the gastrocnemius and soleus muscles and inserts into the posterior surface of the calcaneus.^1-4^ It is covered by a paratenon, which runs externally from its muscular origin to its bony insertion, and its main innervation is provided by the tibial nerve.^5,6^ Despite being the strongest tendon in the human body,^7^ it has the highest prevalence of injury in the lower limbs.^4,3^ Its primary function is to transmit the force produced by the triceps surae to the heel, enabling ankle plantarflexion, as well as to store and release energy as a shock absorber during gait and running.^8^

Rupture of the calcaneal tendon causes a significant functional impact, with a global incidence of 11 to 37 cases per 100,000 individuals per year,^1,3,7^ predominantly in men, with the first peak between 25 and 40 years of age and the second after 60 years, occurring more frequently during physical activity.^1,3,7,9-11^ The etiological factors include reduced tendon vascularization, sports practice in individuals with an unprepared musculoskeletal system, biomechanical foot abnormalities or tendon structural abnormalities, exercise-induced hyperthermia, overweight, decreased strength and/or flexibility of the plantar flexor muscles, and the negative influence of topical corticosteroids and fluoroquinolone antibiotics.^5,12,13^ Mechanisms of injury include resisted plantarflexion, sudden and exaggerated ankle dorsiflexion, and forced dorsiflexion with the joint in plantarflexion.^12^

This type of injury damages the organized architecture of collagen fibers and increases type III collagen content at the site, with a mean tendon elongation of 1.5 to 3.5 cm, while surgical repairs may achieve only up to 1.2 cm of elongation.^3,14^

Diagnosis of acute calcaneal tendon rupture includes patient history, typically reporting a popping sound and/or a sensation of being struck in the posterior leg, and physical examination, which may reveal edema, acute pain, palpable gap, increased passive ankle dorsiflexion, decreased strength of the ankle plantar flexors, inability or difficulty standing on tiptoe, and positive Thompson and Matles tests.^3,5,15,16^ Imaging exams may be performed to confirm rupture, assess its extent and location, and detect tendon pathology.^7^

Treatment of acute calcaneal tendon rupture is divided into two modalities: non-surgical and surgical.^3,9,17,18^ Surgical treatment options described in the literature include open, minimally invasive, and percutaneous techniques.^19,20^

The stages of tissue healing are as follows:

- Inflammatory Response: begins immediately after injury, laceration, or surgical repair and ends within 6 days. The bleeding caused by the rupture leads to hematoma formation and activation of platelets and neutrophils, releasing growth factors, chemotactic factors, and vasoactive mediators.^21,22^ This response triggers fibrin clot formation and consequently stimulates fibroblast activity.^23^ Subsequently, leukocytes and macrophages invade the area to clear cellular and tissue debris.^24^ At this stage, protection of the lesion is necessary, controlling inflammation with rest, immobilization, and elevation to promote healing.^13^
- Repair and Proliferation: begins 48 hours after injury and lasts up to 6–8 weeks. The main agent in this stage is the macrophage, which activates fibroblasts that secrete type III collagen for tendon repair, forming a disorganized collagen matrix with smaller, less resistant fibers that elongate easily.^2,3^ After 10 to 14 days, scar tissue forms at the site joining the ruptured tendon ends. As the process continues, collagen deposition shifts from type III to type I, characterized by greater cross-linking, larger fibrils, and increased strength. In this phase, where the healing tissue is still disorganized and susceptible to reinjury if excessively tensioned, light and pain-free isometric contractions may be initiated. These contractions promote blood circulation, aid fibril organization through mechanical loading, stimulate proper muscle use awareness, and prevent reflex inhibition of immobilized muscle groups.^13^ Isometric contractions performed in a shortened muscle position enhance actin-myosin fiber mobility without overloading the ruptured tissue, while dynamic joint traction or passive compression along the contraction plane allows tendon excursion, promoting healing and reducing adhesion formation. Tendons also heal faster when subjected to mechanical loading, with daily episodes of tensile stress sufficient to stimulate healing without excessive elongation.^25^ Controlled physical activity is a specific physiological stimulus that can enhance functional capacity and reverse disuse atrophy, provided that intensity, frequency, and duration parameters are appropriately managed.^26^
- Remodeling and Maturation: the third healing stage, beginning 1 to 3 months post-injury and lasting for years. This stage is marked by reduced cellularity and synthetic activity, increased organization of the extracellular matrix, and a biochemical profile closer to normal.^2,24,27^ Functional linear alignment of collagen typically appears in the second month, with greater tensile strength.^2,28,29^ Due to the limited neuromuscular control in this phase, controlled forces simulating normal tissue loading are important, and adhesions need to be broken down. Healthy, repetitive loads promote tendon remodeling, improving structure and function. However, the material properties of these scars never fully replicate those of intact tendon, and biomechanical properties may be reduced by up to 30%, even when healing stages are complete, due to the persistent proportion of type III collagen.^28-30^ Thus, repaired tendons do not fully restore their original characteristics, resulting in an altered biological and mechanical environment.

Accordingly, the objective of this study is to present a rehabilitation protocol based on the tendon healing phases, following surgical repair of acute calcaneal tendon rupture, as proposed by the Physiotherapy Department of the Institute of Orthopedics and Traumatology of the Hospital das Clínicas, University of São Paulo (IOT-HCFMUSP).

## MATERIALS AND METHODS

A literature review was conducted over the last 20 years using the following keywords to identify relevant studies within the databases: "Calcaneal Tendon," "Rupture," "Open Surgery," "Minimally Invasive Surgical Procedures," "Physiotherapy," and "Rehabilitation." The search strategies involved first identifying descriptors in the DeCS portal and then applying the descriptors in both Portuguese and English within the PubMed, BVS, and PEDro databases. The keywords "Calcaneal Tendon" and "Rupture" were essential for the relevance of the search results, with the research direction further refined by the complementary terms related to the different types of surgical and therapeutic interventions.

### Protocol

The protocol was developed by the Physiotherapy Department of the Institute of Orthopedics and Traumatology of the Hospital das Clínicas, Faculty of Medicine, University of São Paulo, and is presented in this study to outline the objectives and appropriate interventions for each physiological stage of the lesion within postoperative rehabilitation of the calcaneal tendon.

The first phase comprises the two firsts postoperative weeks, with the objectives of reducing edema, promoting circulation, and maintaining the strength of muscle groups adjacent to the lesion.

### Phase 1

- Positioning/elevation of the lower limb (training in changing decubitus);
- Gait training without weight-bearing on the operated limb;
- Isometric exercises during immobilization;
- Toe flexion-extension exercises;
- Active knee extension exercises in sitting position;
- Straight leg raise in the supine position.

The second phase occurs in the 3rd postoperative week, aiming at gait training, improved activation, maintenance, and strengthening of the lower limb muscles. At this stage, dorsiflexion is not permitted, in order to avoid placing tension on the suture site.

### Phase 2

- Gait training with immobilizing orthosis (progressive load tolerated by the patient);
- Isometric strengthening of invertors, evertors, and plantar flexors without joint movement, to be performed while immobilized;
- Free active toe flexion-extension exercises during immobilization;
- Straight leg raises with load in the supine, lateral, and prone positions;
- Active knee extension with load in sitting position.

The 3rd phase covers the period from the 4th to the 6th postoperative week, with the objective of protecting the healing process, joint mobilization, strengthening of ankle and foot muscles, and progressive strengthening of the lower limbs. At this stage, ranges of motion of plantar flexion, inversion, and eversion are allowed, returning only to the neutral position to protect the sutured area.

### Phase 3

- Straight leg raises with load in the supine, lateral, and prone positions with progressive load increase;
- Active joint mobilization of plantar flexion, inversion, and eversion with return to neutral position;
- Active resisted strengthening with elastic bands for plantar flexion, inversion, and eversion with progressive load increase. **Do not perform exercises for dorsiflexors;**
- Soleus strengthening in the sitting position, with the foot placed forward of the knee (knee flexion angle less than 90°).

The 4th phase, occurring between the 8th and 12th postoperative weeks, aims to achieve full ankle range of motion and progressive lower limb strengthening, in addition to improving cardiorespiratory condition. Removal of the orthosis heel lift, when indicated, and discontinuation of immobilization will occur according to medical clearance.

### Phase 4

- Straight leg raises with load in the supine, lateral, and prone positions with progressive load increase;
- Active dorsiflexion mobilization;
- Maintenance of active resisted strengthening with elastic bands for plantar flexion, inversion, and eversion, with initiation of dorsiflexor strengthening;
- Stationary cycling;
- Squats;
- Sensorimotor training.

The 5th phase begins after completion of 12 weeks, aiming to achieve full ankle mobility, posterior chain stretching, strengthening of the triceps surae, improvement of balance and gait, and training for preparatory jumping movements. At this stage, posterior chain stretching is permitted while keeping the ankle in neutral position, protecting stress on the sutured tendon. Sensorimotor control training aims to prepare the patient for return to sports practice.

### Phase 5

- Progressive strengthening of the triceps surae on a step, with concentric and eccentric bipodal exercises, progressing to unipodal;
- Protected posterior chain stretching;
- Advanced sensorimotor control training with direction changes;
- Bipodal plyometrics progressing to unipodal, according to the patients ability.

## DISCUSSION

The purpose of this study was to present the rehabilitation protocol of the Physiotherapy Department of IOT-HCFMUSP and to compare it with findings from the literature based on a bibliographic review.

Maquirriain J. reported that early tension and weight-bearing on a repaired tendon improved muscle strength and tendon vascularization. Suchak et al. compared early weight-bearing beginning 2 weeks after repair with weight-bearing allowed only after 6 postoperative weeks.^31,32^ At the sixth week, the group that underwent early weight-bearing presented significantly better scores in the domains of physical and social functionality, emotional health, and vitality in a quality-of-life questionnaire, in addition to reporting fewer limitations in daily activities. At six months postoperatively, no significant differences were observed between the groups in any outcome, and both showed low muscle strength in the triceps surae. It is noteworthy that there was no rerupture in either group. Based on this evidence, early weight-bearing is adopted as an important part of the proposed protocol.

Immobilization with an orthosis is used to avoid early dorsiflexion and thus prevent stretching of the suture at the calcaneal tendon,^31,33^ Kisner et al. warned that early or excessive mobilization may damage the injured tissue, meaning that the range of motion should not be performed when it negatively interferes with the healing process.^13,31^ Bevoni et al, stated that dorsiflexion is permitted up to 5° at 6 postoperative weeks. At this stage, type I collagen production increases and the tendon callus reaches its largest size. Although the tissue is more fragile at this point, the greater transverse area of the callus compensates for its weaker composition.^9^ In the proposed protocol, dorsiflexion is initiated at the 8th week, without range restrictions except for patient tolerance, since it is functionally unfeasible to control dorsiflexion degrees at minimal ranges during home-based exercises without therapist supervision.^33^

Rosenzweig e Azar describe that the foot is placed in the plantigrade position 4 to 6 weeks after repair, without specifying the moment when dorsiflexion beyond neutral is permitted. They recommend that the patient use an equinus positioning splint from the 2nd to 4th weeks, a removable orthosis allowing only plantar flexion from the 6th to 8th weeks, and an orthosis with a lock in the neutral position starting from the 12th week, until the patient presents at least 80% muscle strength of the contralateral limb and an unspecified ROM.^34^ In the proposed protocol, the immobilizing orthosis is introduced at the 2nd week and removed within 12 weeks at the physician's discretion. From this period onward, the patient is allowed to perform ankle dorsiflexion and propulsion through triceps surae activation during gait, consistent with Medeiros et al., who state that wound resistance reaches 80% of the original tissue strength after 3 months.^35^

Direct stretching of the calcaneal tendon is not allowed because, as cited by Maquirriain J., creep is a mechanical property in which a constant load causes lengthening over time, leading to tension loss in the initial postoperative phase. Therefore, tendon stretching results in morbidity and triceps surae weakness at the end of movement, impairing rehabilitation.^31^

Suchak et al. also applied an accelerated rehabilitation method in which the neutral position of the operated ankle was already permitted between the 2nd and 3rd postoperative weeks, concurrently with the allowance of active dorsiflexion, apparently without restriction in the maximum angle reached by the patient.^32^ In the proposed protocol, strengthening of plantar flexors, invertors, and evertors is allowed from the 4th postoperative week, since these movements do not place tension on the sutured tendon. At the 8th week, exercise progression is initiated, along with the release of ankle dorsiflexion and strengthening of the triceps surae with bipodal support, as by this time the tendon repair and proliferation healing phase is completed. At this stage, type I collagen deposition begins and accelerates, establishing a biomechanical and biochemical profile closer to the physiological tissue.

According to Maquirriain J., it is important to maintain or increase contraction strength of the muscles of the operated lower limb, with emphasis on the triceps surae, thereby reducing the risk of injury and preventing stretching of the calcaneal tendon, which is consistent with the approach proposed in this protocol. Another essential aspect is sensorimotor training, given that patients with calcaneal tendon injury present deficits in this area.^31^

The time to return to sports practice coincides with findings in the literature and with the protocol developed by this group. It is recommended that return to prior sports activity occur 5 to 6 months after surgery. However, for this return to be adequate, it requires prior preparation promoted by rehabilitation.^13,33,34,36^ Postoperative rehabilitation guidelines for calcaneal tendon repair published by the Department of Sports Medicine of the University of Wisconsin consider patients eligible to return to sport-specific activities from the 16th postoperative week, through exercises simulating sports activities, similar to the protocol proposed here.^33^

A protocol similar to the one proposed was identified, in terms of exercise progression, weight-bearing permission, use of assistive devices, release of dorsiflexion movement, and return to sport-specific training.^36^ However, this approach was applied to the postoperative period of a modified open surgical technique, associated with a gastrocnemius tendon flap and deep posterior compartment fasciotomy. Outcomes were satisfactory in both surgical techniques, as patients presented no differences between the operated and contralateral limbs in functional test results, and no long-term postoperative failures were observed. This evidence shows that, despite different surgical techniques, the proposed rehabilitation protocol presents minimal risk of damage to the surgical site.

## References

[1] Park SH, Lee HS, Young KW, Seo SG (2020). Treatment of Acute Achilles Tendon Rupture. Clin Orthop Surg.

[2] Ramos DM, Abdulmalik S, Arul MR, Sardashti N, Banasavadi-Siddegowda YK, Nukavarapu SP (2022). Insulin-Functionalized Bioactive Fiber Matrices with Bone Marrow-Derived Stem Cells in Rat Achilles Tendon Regeneration. ACS Appl Bio Mater.

[3] King CM, Vartivarian M (2023). Achilles Tendon Rupture Repair: Simple to Complex. Clin Podiatr Med Surg.

[4] Pierre-Jerome C, Moncayo V, Terk MR (2010). MRI of the Achilles tendon: a comprehensive review of the anatomy, biomechanics, and imaging of overuse tendinopathies. Acta Radiol.

[5] Kitaoka HB (2005). Master techniques in orthopaedic surgery: tornozelo e pé.

[6] MacMahon A, Deland JT, Do H, Soukup DS, Sofka CM, Demetracopolous CA, DeBlis R (2016). MRI Evaluation of Achilles Tendon Rotation and Sural Nerve Anatomy: Implications for Percutaneous and Limited-Open Achilles Tendon Repair. Foot Ankle Int.

[7] Geng X, Yang XG, Teng ZL, Hu XX, Wang C, Zhang C (2023). Is a Preoperative MRI Scan Necessary for Acute Achilles Tendon Rupture?. Orthop Surg.

[8] Pang BS, Ying M (2006). Sonographic measurement of achilles tendons in asymptomatic subjects: variation with age, body height, and dominance of ankle. J Ultrasound Med.

[9] Correia MST (2022). Abordagem aberta VS percutânea. [Tese].

[10] Del Buono A, Volpin A, Maffulli N (2014). Minimally invasive versus open surgery for acute Achilles tendon rupture: a systematic review. Br Med Bull.

[11] Huttunen TT, Kannus P, Rolf C, Felländer-Tsai L, Mattila VM (2014). Acute achilles tendon ruptures: incidence of injury and surgery in Sweden between 2001 and 2012. Am J Sports Med.

[12] Khan RJ, Fick D, Keogh A, Crawford J, Brammar T, Parker M (2005). Treatment of acute achilles tendon ruptures. A meta-analysis of randomized, controlled trials. J Bone Joint Surg Am.

[13] Kisner C, Colby LA (2009). Exercícios Terapêuticos - Fundamentos e Técnicas.

[14] Krapf D, Kaipel M, Majewski M (2012). Structural and biomechanical characteristics after early mobilization in an Achilles tendon rupture model: operative versus nonoperative treatment. Orthopedics.

[15] Chiodo CP, Glazebrook M, Bluman EM, Cohen BE, Femino JE, Giza E (2010). Diagnosis and treatment of acute Achilles tendon rupture. J Am Acad Orthop Surg.

[16] Barros TEP, Lech O (2002). Exame Físico em Ortopedia.

[17] Wallace RG, Traynor IE, Kernohan WG, Eames MH (2004). Combined conservative and orthotic management of acute ruptures of the Achilles tendon. J Bone Joint Surg Am.

[18] Ecker TM, Bremer AK, Krause FG, Müller T, Weber M (2016). Prospective Use of a Standardized Nonoperative Early Weightbearing Protocol for Achilles Tendon Rupture: 17 Years of Experience. Am J Sports Med.

[19] Li Q, Wang C, Huo Y, Jia Z, Wang X (2016). Minimally invasive versus open surgery for acute Achilles tendon rupture: a systematic review of overlapping meta-analyses. J Orthop Surg Res.

[20] Carmont MR, Rossi R, Scheffler S, Mei-Dan O, Beaufils P (2011;). Percutaneous & Mini Invasive Achilles tendon repair. Sports Med Arthrosc Rehabil Ther Technol.

[21] Aspenberg P (2007). Stimulation of tendon repair: mechanical loading, GDFs and platelets. A mini-review. Int Orthop.

[22] Kajikawa Y, Morihara T, Watanabe N, Sakamoto H, Matsuda K, Kobayashi M (2007). GFP chimeric models exhibited a biphasic pattern of mesenchymal cell invasion in tendon healing. J Cell Physiol.

[23] Maxey L, Magnusson J (2003). Reabilitação pós-cirúrgica para o paciente ortopédico.

[24] Sharma P, Maffulli N (2006). Biology of tendon injury: healing, modeling and remodeling. J Musculoskelet Neuronal Interact.

[25] Andersson T, Eliasson P, Hammerman M, Sandberg O, Aspenberg P (2012). Low-level mechanical stimulation is sufficient to improve tendon healing in rats. J Appl Physiol (1985).

[26] Almeida CS, Greve JMD (2007). Tratado de Medicina de Reabilitação.

[27] Lin TW, Cardenas L, Soslowsky LJ (2004). Biomechanics of tendon injury and repair. J Biomech.

[28] Oliva F, Via AG, Maffulli N (2011). Role of growth factors in rotator cuff healing. Sports Med Arthrosc Rev.

[29] Wang JH (2006). Mechanobiology of tendon. J Biomech.

[30] Andarawis-Puri N, Flatow EL, Soslowsky LJ (2015). Tendon basic science: Development, repair, regeneration, and healing. J Orthop Res.

[31] Maquirriain J (2011). Achilles tendon rupture: avoiding tendon lengthening during surgical repair and rehabilitation. Yale J Biol Med.

[32] Suchak AA, Bostick GP, Beaupré LA, Durand DC, Jomha NM (2008). The influence of early weight-bearing compared with non-weight-bearing after surgical repair of the Achilles tendon. J Bone Joint Surg Am.

[33] Bevoni R, Angelini A, D'Apote G, Berti L, Fusaro I, Ellis S, Schuh R, Girolami M (2014). Long term results of acute Achilles repair with triple-bundle technique and early rehabilitation protocol. Injury.

[34] Rosenzweig S, Azar FM (2009). Open repair of acute Achilles tendon ruptures. Foot Ankle Clin.

[35] Medeiros AC, Dantas AM (2017). Cicatrização das feridas cirúrgicas. Journal of Surgical and Clinical Research.

[36] Ozer H, Selek HY, Harput G, Oznur A, Baltaci G (2016). Achilles Tendon Open Repair Augmented With Distal Turndown Tendon Flap and Posterior Crural Fasciotomy. J Foot Ankle Surg.

